# Population pharmacokinetics of everolimus in patients with seizures associated with focal cortical dysplasia

**DOI:** 10.3389/fphar.2023.1197549

**Published:** 2023-11-24

**Authors:** Jinha Park, Se Hee Kim, Jongsung Hahn, Hoon-Chul Kang, Sang-Guk Lee, Heung Dong Kim, Min Jung Chang

**Affiliations:** ^1^ Department of Pharmaceutical Medicine and Regulatory Science, Colleges of Medicine and Pharmacy, Yonsei University, Seoul, Republic of Korea; ^2^ Department of Pharmacy, College of Pharmacy, Yonsei Institute of Pharmaceutical Sciences, Yonsei University, Seoul, Republic of Korea; ^3^ Pediatric Neurology, Department of Pediatrics, Yonsei University College of Medicine, Severance Children’s Hospital, Epilepsy Research Institute, Seoul, Republic of Korea; ^4^ Department of Pharmacy, Jeonbuk National University, Jeonju, Republic of Korea; ^5^ Department of Laboratory Medicine, Severance Hospital, Yonsei University College of Medicine, Seoul, Republic of Korea; ^6^ Department of Pediatrics, Kangbuk Samsung Hospital, Sungkyunkwan University, Seoul, Republic of Korea; ^7^ Graduate Program of Industrial Pharmaceutical Science, Yonsei University, Incheon, Republic of Korea

**Keywords:** everolimus, focal cortical dysplasia, epilepsy, population pharmacokinetics, non-linear mixed-effect modeling

## Abstract

**Background:** Everolimus is an inhibitor of mammalian target of rapamycin complex 1. As mutations in *TSC1* and *TSC2*, which cause partial-onset seizures associated with TSC, were found in focal cortical dysplasia type Ⅱ (FCD Ⅱ) patients, a clinical trial has been performed to explore the efficacy and safety of everolimus in FCD patients. However, no dosage regimen was determined to treat FCD II. To recommend an optimal dose regimen for FCD patients, a population pharmacokinetic model of everolimus in FCD patients was developed.

**Methods:** The data of everolimus were collected from September 2017 to May 2020 in a tertiary-level hospital in Korea. The model was developed using NONMEM^®^ software version 7.4.1 (Icon Development Solutions, Ellicott City, MD, United States).

**Results:** The population pharmacokinetics of everolimus was described as the one-compartment model with first-order absorption, with the effect of BSA on clearance. The final model was built as follows: TVCL = 12.5 + 9.71 × (BSA/1.5), TVV = 293, and TVKA = 0.585. As a result of simulation, a dose higher than 7 mg/m^2^ is needed in patients with BSA 0.5 m^2^, and a dose higher than 6 mg/m^2^ is needed in patients with BSA 0.7 m^2^. A dose of 4.5 mg/m^2^ is enough in the population with BSA higher than 1.5 m^2^ to meet the target trough range of 5–15 ng/mL.

**Conclusion:** Based on the developed pharmacokinetics model, the optimal dose of everolimus in practice was recommended by considering the available strengths of Afinitor disperz^®^, 2 mg, 3 mg, and 5 mg.

## 1 Introduction

Everolimus is an inhibitor of mammalian target of rapamycin complex 1 (mTORC1), which play a role as an immunosuppressive and antineoplastic drug indicated for various organ transplantations and tumors. It inhibits the interaction between mTORC1 and FK506-binding protein-12 (FKBP-12) by binding to FKBP-12 with high affinity (Gabriele et al., 2004; Sánchez-Fructuoso, 2008). The downstream signaling related to cell cycle and glycolysis is altered, and the growth of tumor is inhibited consequently.

Based on the etiology of the tuberous sclerosis complex (TSC), which is related to mutations in oncogene suppressor *TSC1* and *TSC2* genes causing overactivation of mTOR, a randomized clinical trial examining Everolimus in a Study of Tuberous Sclerosis Complex (EXIST-3) was performed to evaluate the efficacy and safety of everolimus as an adjuvant treatment for seizures in TSC patients (French et al., 2016). As a result, in 2018, Afinitor disperz^®^ (Novartis Pharmaceuticals Corporation) was approved for adjunctive treatment of adults and pediatrics older than 2 years with partial-onset seizure associated with TSC. The initial dose regimen was decided by age and the presence of the concomitant CYP3A4/P-glycoprotein inducer, which ranged from 3 mg/m^2^ to 6 mg/m^2^ once daily. The efficacy of reducing the frequency of seizures was shown in both low and high target trough concentration ranges, 3–7 ng/mL and 9–15 ng/mL, respectively.

Focal cortical dysplasia (FCD), which is characterized by abnormal development of the cerebral cortex, is one of the most important causes of refractory epilepsy which does not respond to conventional antiepileptic drugs in pediatrics (Sanjay et al., 2009). FCD is classified into three types by neuropathological features: FCD Ⅰ presenting radial- and/or tangential-shaped dyslamination of the cortex, FCD Ⅱ presenting cortical dyslamination and dysmorphic neurons, and FCD Ⅲ related to other brain lesions (Blümcke et al., 2011).

There are many studies showing that FCD Ⅱ is related to the hyperactivation of the mTOR pathway (Baybis et al., 2004; Miyata et al., 2004; Ljungberg et al., 2006). Furthermore, in 2017, mutations in *TSC1* and *TSC2* that cause partial-onset seizures associated with TSC were found in FCD Ⅱ patients (Lim et al., 2015; Lim et al., 2017). Recently, a pilot study to determine the safety and mechanism of the action of everolimus in patients with TSC and FCD was reported (Fouladi et al., 2007; Dirk Jan et al., 2012; de Wit et al., 2016; Leitner et al., 2022). This allows us to hypothesize that everolimus, an mTOR inhibitor, can be used as a treatment of refractory seizures associated with FCD. The pharmacokinetics (PK) of everolimus was reported using both one- and two-compartment models (Atsuko Tanaka et al., 2016; Combes et al., 2018; Dirk Jan et al., 2012)). A population PK study based on EXIST-1, -2, and -3 reported that a two-compartment model with first-order absorption and body surface area showed that CYP3A or P-gp inducers increased the clearance of everolimus (Combes et al., 2018). However, no PK was reported in patients with FCD II, and no dosage regimen has been determined yet.

The purpose of this study is to develop a population pharmacokinetic model of everolimus in patients with seizures associated with focal cortical dysplasia (FCD) type Ⅱ, analyzing clinical covariates to suggest an optimal dose regimen for this population.

## 2 Materials and methods

### 2.1 Study design and population

This analysis was performed with data available from a double-blinded crossover randomized clinical trial conducted at Severance Hospital in Seoul, Republic of Korea, from 2017 to 2020. The study protocol was approved by the Institutional Review Board (IRB NO. 4-2017-0299) of Severance Hospital. To obtain everolimus concentrations drawn at the time after dose from January 2020 to May 2020, an additional study protocol was approved by the Institutional Review Board (IRB NO. 4-2019-1232) of Severance Hospital. All participants and the legal surrogates provided written informed consent which explained the purpose and details of the study.

The inclusion and exclusion criteria for participants are described in the Supplementary Material. The clinical trial consists of the following four phases: baseline for 4 weeks, core Ⅰ for 12 weeks, core Ⅱ for 12 weeks, and extension phase for 29 weeks. After screening in the baseline phase for 4 weeks, patients were assigned randomly to one of two groups: everolimus (Afinitor disperz^®^, tablet for oral suspension) or placebo. After being administered with everolimus or placebo for core phase Ⅰ, patients received the other treatment, by crossover, for core phase Ⅱ. Only patients who agreed to participate in the extension phase were administered with everolimus until the completion of the trial.

### 2.2 Drug dosage and data collection

The initial dose of everolimus was 4.5 mg/m^2^/day, which was used as an effective and safe dose in the EXIST-1 study (Franz et al., 2013). The target range of trough concentration was 5–15 ng/mL, and dose adjustment was performed through TDM for patients with a trough concentration lower or higher than the target range. The increase or decrease in the adjusted dose was 2 mg. Patients were administered with everolimus (Afinitor disperz^®^, tablet for oral suspension) once daily at the set time. Patients were also administered with more than one antiepileptic drug concomitantly. The specific products and doses of the concomitant antiepileptic drugs were not changed during the baseline and core phases.

The following data were collected from September 2017 to May 2020: the amount of everolimus administered, the actual time of administration, the actual time of sampling, the concentrations of everolimus, age, sex, weight (kg), body surface area (BSA, m^2^), serum creatinine (mg/dL), ALT (IU/L), AST (IU/L), hemoglobin (g/dL), hematocrit (%), RBC (10^6^/μL), albumin (g/dL), and the presence of concomitant CYP3A4 inducer and inhibitor (strong, moderate, and weak) (Supplementary Table S1).

### 2.3 Sampling strategy and bioanalysis

Blood samples for therapeutic drug monitoring were collected before administration and at weeks 2, 3, 4, and 8 in core phase Ⅰ, at weeks 14, 15, 16, and 20 in core phase Ⅱ, and at weeks 25, 28, and 40 in the extension phase. Additional blood samples were collected at any time from 1 to 4 h after dose at weeks 24, 28, or 40 in the extension phase. Each drawn sample was 1 mL or 2 mL in volume for patients under or over the age of 6 years, respectively. Immediately after collection, the samples were placed in EDTA tubes.

The concentrations of everolimus were measured by validated high-performance liquid chromatography/tandem mass spectrometry (HPLC/MS/MS) using an Agilent 1260 (Agilent Technologies, CA, United States) coupled with an API 4000 (Sciex, Concord, Ontario, Canada). Then, 50 μL of deionized water and 50 μL of 0.1 M ZnSO_4_ were added to 50 μL of sample, and the mixture was vortexed for 15 s. 5 μL each of ascomycin, sirolimus-d3, cyclosporin D, and everolimus-d4 as an internal standard and 130 μL of methanol were added to the mixture. After vortexing for 60 s, the mixture was centrifuged at 14,000 rpm for 10 min. Chromatographic separation was performed on a Guard column and a C_8_ column (4 × 2.0 mm and 10 × 2.0 mm, respectively, Phenomenex, Torrance, CA, United States) with solvent A (deionized water with 2 mM ammonium acetate and 0.1% formic acid) and solvent B (100% methanol with 2 mM ammonium acetate and 0.1% formic acid) as the mobile phase. The gradient was as follows: 50:50 v/v for the first 0.1 min; 100% B for the next 1.1 min; and 50:50 v/v for the last 1.8 min. The flow rate was 650 μL/min for 3 min. The lower limit of quantification for everolimus was 1.1 ng/mL. The assay was validated within the range 1.1–41.6 ng/mL. The inter- and intra-assay coefficients of variation were below 7.3%.

### 2.4 Population pharmacokinetic analysis

Population pharmacokinetic modeling was conducted using the first-order conditional estimation method with the eta–epsilon interaction (FOCE + I) algorithm in NONMEM^®^ software version 7.4.1 (Icon Development Solutions, Ellicott City, MD, United States) assisted by Perl-speaks-NONMEM (PsN, version 4.7.0), Pirana (version 2.9.7, Certara, NJ, United States), and Xpose4 (version 4.6.1) embedded in R (version 3.5.1; http://www.r-project.org/).

One- and two-compartmental PK models with first-order absorption were evaluated for a potential structural model using subroutines ADVAN2 TRANS2 and ADVAN4 TRANS4 in the NONMEM library, respectively. Inter-individual variability (η) was estimated with an exponential error model as follows:

$$\theta_{i}=\theta_{\text{POP}}\times \exp\left(\eta_{i}\right),$$

where θ_i_ is the individual PK parameter for the i^th^ individual, θ_POP_ is the population PK parameter, and η_i_ is a random variable of PK parameter which is assumed to follow a log-normal distribution with a mean of zero and a variance of ω^2^.

Intra-individual variability (ε) was estimated with an additive, proportional, and a combined model as follows:

$$C_{\text{ij}}=C_{\text{predij}}+\varepsilon_{\text{ij},\text{additive}},$$

$$C_{\text{ij}}=C_{\text{predij}}\times \left(1+\varepsilon_{\text{ij},\text{proportional}}\right),$$

$$C_{\text{ij}}=C_{\text{predij}}\times \left(1+\varepsilon_{\text{ij},\text{proportional}}\right)+\varepsilon_{\text{ij},\text{additive}},$$

where C_ij_ is the j^th^ observed concentration for the i^th^ individual, C_predij_ is the corresponding predicted concentration, and ε_ij,additive_ and ε_ij,proportional_ are random variables which are assumed to follow a normal distribution with a mean of zero and a variance of σ^2^. The best fitted base model was selected based on the minimum of the objective function value (OFV), which is statistically equivalent to the −2log likelihood, visual inspection on the basic goodness of fit plots, and the plausibility of relative standard errors. The criterion of a statistically significant decrease in OFV was 3.84 (χ^2^ distribution, degrees of freedom = 1, *p*-value<0.05). The basic goodness-of-fit plots comprised four types of plots: observed concentration *versus* individual predicted concentration, observed concentration *versus* population predicted concentration, conditional weighted residuals (CWRES) *versus* population predicted concentration, and conditional weighted residuals (CWRES) *versus* time. Individual concentration–time plots and the eigenvalue of the models were also explored.

To explain the inter-individual variability of PK parameters, the following 14 potential covariates were evaluated: age (years), sex (0 for male; 1 for female), weight (kg), BSA (m^2^), serum creatinine (mg/dL), ALT (IU/L), AST (IU/L), hemoglobin (g/dL), hematocrit (%), RBC (10^6^/μL), albumin (g/dL), and the presence of at least one concomitant CYP3A4 strong inducer, weak inducer, and weak inhibitor (0 for absence; 1 for presence).

Continuous covariates (age, weight, BSA, serum creatinine, ALT, AST, hemoglobin, hematocrit, RBC, and albumin) centered on their median values were tested using linear, exponential, power, and proportional models. Moreover, for categorical covariates (sex and the presence of at least one concomitant CYP3A4 inducer or inhibitor), linear, exponential, power, and proportional models were used.

Covariates were analyzed using a stepwise method which consists of a forward selection step and a backward elimination step. In the forward selection step, a covariate was selected if the OFV of the model with added covariate decreased under 3.84 (χ^2^-test, *p*-value<0.05) compared to the prior model. The procedure was performed until no more covariate reduced the OFV of the model significantly, and the full model was constructed with all influential covariates. In the backward elimination step, the covariate was retained in the final model if the OFV increased over 6.63 (χ^2^-test, *p*-value<0.01) when each of the included covariates was deleted one by one. A decision on covariate selection was made at each step based on biological and clinical plausibility.

### 2.5 Final model validation

Validation of the final model was performed to evaluate its accuracy and robustness through bootstrap, goodness-of-fit plot, and prediction-corrected visual predictive check (pc-VPC). For bootstrap, 5,000 sets of data were resampled to validate the final model internally by comparing to estimates from the final model. Median values of estimates were calculated, and 95% confidence intervals were constructed by 2.5^th^ and 97.5^th^ percentiles from bootstrap results. Goodness-of-fit plots were examined to evaluate the fitting of predictions to observations. PC-VPC was performed with 1,000 simulations to diagnose the predictive performance of the final model graphically. Median, 2.5^th^, and 97.5^th^ percentiles were visually assessed with 95% confidence interval.

### 2.6 Simulation

To predict the concentrations of everolimus at steady state using influential covariates, Monte Carlo simulation was performed based on the constructed final model. Dosages of 3 mg/m^2^, 4.5 mg/m^2^, 5 mg/m^2^, 6 mg/m^2^, 7 mg/m^2^, and 9 mg/m^2^ once daily were simulated for each BSA of 0.5 m^2^, 1 m^2^, 1.5 m^2^, 1.7 m^2^, and 2 m^2^, which were the extracted values from the baseline range of patients in this study. Additionally, 3 mg/m^2^, 4.5 mg/m^2^, 5 mg/m^2^, 6 mg/m^2^, and 7 mg/m^2^ were simulated for the groups with BSA 0.7 m^2^ to explore the optimized dose regimen for FCD patients. A total of 35 scenarios are shown in Supplementary Table S2. Concentrations were generated for 1,000 subjects in each scenario assuming that concentrations of everolimus reach a steady state after 14 days.

## 3 Results

### 3.1 Patient characteristics

The demographics of the 22 total patients are described in Table 1. Continuous variables are shown in median with ranges as minimum, interquantile, and maximum. Categorical variables are shown in the numbers of patients, with portions in percentage. The median age was 13.5 years (range 4–32 years), and nine male patients (40.9%) were included. The median BSA was 1.5 m^2^ (range 0.6–2 m^2^), and the median albumin was 4.6 g/dL (range 4.1–5.2 g/dL). Only one patient was administered with CYP3A4 strong inducers concomitantly during this study, and another patient who was administered with a CYP3A4 moderate inducer concomitantly dropped out without valid everolimus concentrations.

**TABLE 1:** Baseline characteristics of study patients (n = 22).

Continuous variable	Median	IQR	Min., max.
Age (years)	13.5	12–17.75	4, 32
Body weight (kg)	50	35.5–60	13, 86
Body surface area (m^2^)	1.5	1.23–1.7	0.6, 2
Serum creatinine (mg/dL)	0.67	0.48–0.76	0.33, 0.92
ALT (IU/L)	11	9–13	5, 67
AST (IU/L)	16	14–18	10, 44
Hemoglobin (g/dL)	13.15	12.7–14.3	10.3, 16.5
Hematocrit (%)	38.95	37.8–41.9	35, 47.8
RBC (10^6^/mcL)	4.48	4.25–4.76	3.89, 5.19
Albumin (g/dL)	4.6	4.3–5	4.1, 5.2
Categorical variables	Number	Portion (%)	
Male	9	40.9	
CYP 3A4 inducer^a^			
Strong	1	4.5	
Moderate	0	0	
Weak	14	63.6	
CYP 3A4 inhibitor^a^			
Strong	2	9	
Moderate	1	4.5	
Weak	14	63.6	

ALT, alanine transaminase; AST, aspartate aminotransferase; CYP, cytochrome P450 enzyme; IQR, inter quantile range; min., minimum; max., maximum; RBC, red blood cell.

^a^ The number of patients taking at least one CYP3A4 inducer/inhibitor with everolimus concomitantly.

### 3.2 Population pharmacokinetic model

A total of 152 observed everolimus concentrations of time after dose from 22 patients were included in the population PK modeling. The observed everolimus data were best described by the one-compartment model with the first-order absorption model. The clearance, volume of distribution, and absorption rate of the population were estimated as 21.9 L/h, 302 L, and 0.573 h^−1^, respectively. The inter-individual variability of clearance (ω_CL_^2^) and volume of distribution (ω_V_^2^) were estimated as 0.0409 and 0.0602, which are correlated to 20.2% and 24.5% of the coefficient of variation, respectively. The inter-individual variability of the absorption rate (ω_KA_^2^) was fixed as zero. The residual variability (σ^2^) was best explained by the proportional error model. The objective function value of the base model was 397.372.

All collected covariates were tested in a stepwise manner. For the first forward selection step, the linear model of BSA on clearance was selected, with the largest reduction of OFV (ΔOFV = −6.356). According to the clinical pharmacology review of the FDA (CDER, 2009), the clearance of everolimus increased linearly with BSA, which aligns well with this study. In the second step, the proportional model of albumin on clearance was selected, with a reduction of OFV (ΔOFV = −4.941). This can be explained by the protein-binding portion (75%) of everolimus. The proportional model of RBC on clearance and the power model of RBC on volume of distribution also reduced the OFV (ΔOFV = −5.6 and −4.095, respectively); however, RBC was excluded as a covariate because the bioassay of everolimus was performed with whole blood, meaning that RBC does not influence everolimus concentrations in this study. As a result, the full model had BSA and albumin on clearance.

When BSA was eliminated from the full model, OFV increased significantly (ΔOFV = 6.92), while the removal of albumin from the full model increased OFV to 4.941. The final PK model was constructed with BSA on clearance.

The estimated values of the parameters from the final population PK model are summarized in Table 2 with the medians derived from 5,000 bootstrapped samples in 95% confidence intervals. The estimated values were similar to the median values from the bootstrap result, within 95% confidence interval. The relative standard errors (RSEs) of the random effects were acceptable. All eta shrinkage values were under 40, except IIV of the absorption rate, which was fixed at zero.

**TABLE 2:** Parameter estimates and bootstrap confidence interval.

	Structural model (RSE%^b^) [shrinkage%]	Final model		
		TVCL = CL+ θ_BSA on CL_ × (BSA/1.5) TVV = VTVKA = KA		
		Final model (RSE%^a^) [shrinkage%]	Bootstrap (5,000 replicates)	
			Median	95% CI^b^ (2.5%–97.5%)
Fixed effects				
CL (L/h)	21.9 (16)	12.5 (26)	12.04	5.46, 23.38
V (L)	302 (34)	293 (30)	287.7	72.0, 1454
KA (/h)	0.573 (34)	0.585 (30)	0.594	0.083, 1.195
θ_BSA on CL_	-	9.71 (33)	10.14	2.29, 26.54
Random effects				
Inter-individual variability (ω)				
ω_CL_	0.2022 (51) [13]	0.1652 (47) [15]	0.1565	0.0728, 0.4535
ω_V_	0.2454 (72) [38]	0.2083 (63) [39]	0.1977	0.0539, 1.2360
Residual variability (σ)				
σ _proportional_	0.2922 (15) [7]	0.2943 (14) [6]	0.2888	0.2249, 0.3303
OFV				
OFV _Base model_		OFV _Final model_		Reduction of OFV
397.372		391.016		6.356

BSA, body surface area; CI, confidence interval; CL, clearance; KA, absorption rate; RSE, relative standard error; V, volume of distribution.

^a^ RSE% = (standard error/parameter estimate) × 100.

^b^ 95% CI estimated from 5,000 resampled datasets by using the final population pharmacokinetic model.

**FIGURE 1:**
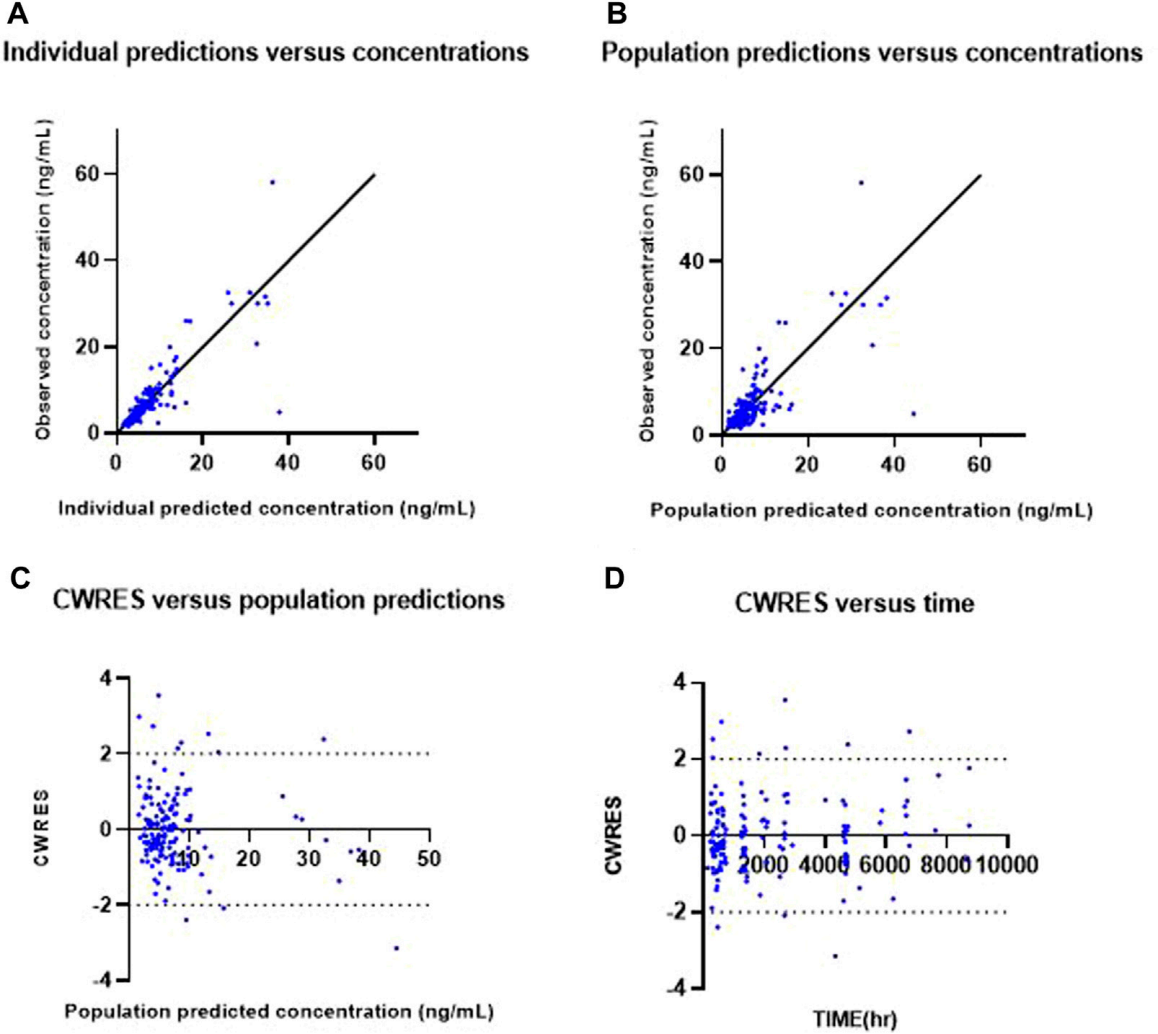
Goodness-of-fit plots of the final model. Observed everolimus concentration *versus* individual predicted (IPRED) concentration **(A)** and *versus* population predicted concentration (PRED) **(B)**. Conditional weighted residuals (CWRES) *versus* population predicted concentration **(C)** and *versus* time **(D)**.

Goodness-of-fit plots for the final PK model are shown in Figure 1. Individual predicted concentration (IPRED, (a)) and population predicted concentration (PRED, (b)) were uniformly distributed near the line, showing that observations equal predictions. Conditional weighted residuals (CWRES) by PRED (c) and time (d) were randomly distributed around zero, without specific trends.

**FIGURE 2:**
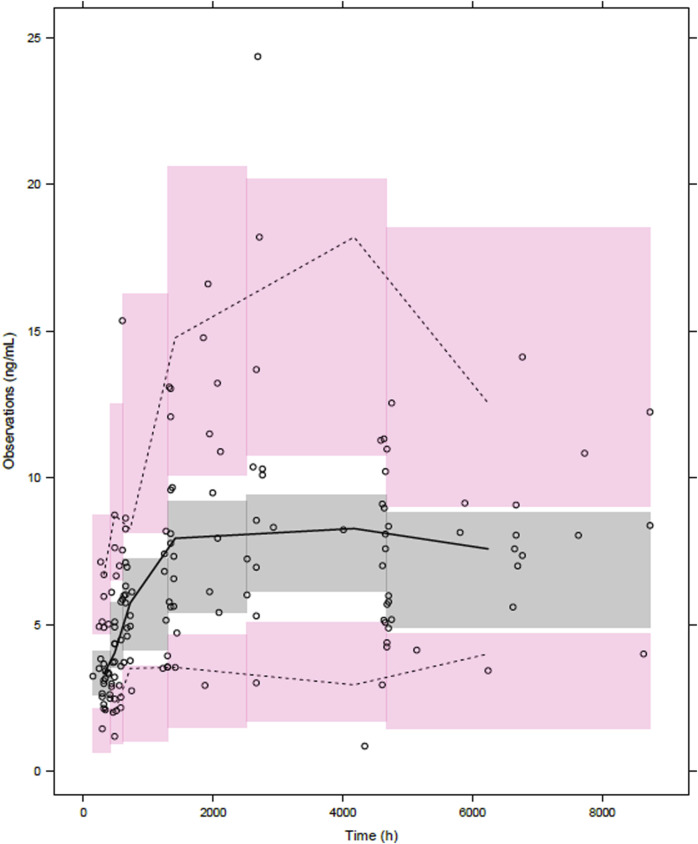
Prediction-corrected visual predictive check of the final model. Open circles, observed everolimus concentrations; solid line, median; lower and upper dashed lines, the 2.5th and 97.5th percentiles of the simulated data, respectively; and shaded areas, 95% confidence intervals for simulated predicted median, 2.5th percentile, and 97.5th percentile constructed from 1,000 simulated datasets of individuals from the original dataset.

The pc-VPC plot for the final PK model, which describes the 2.5th, median and 97.5th observed concentrations by lines, and each corresponding 95% confidence interval for the predicted estimates as given by the shaded areas are shown in Figure 2. Each line is well included in the shaded area, which was constructed from 1,000 simulated datasets from the final population PK model.

### 3.3 Simulations

**FIGURE 3:**
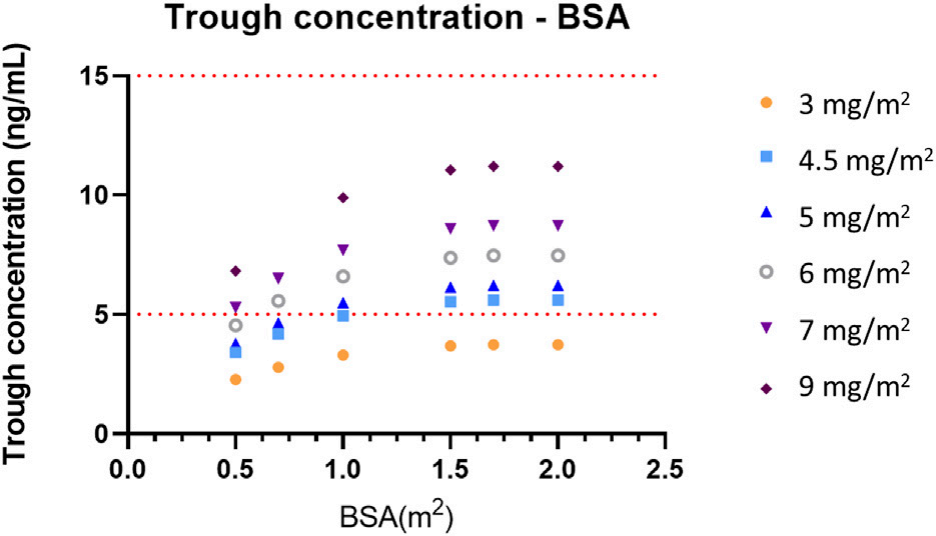
Simulated trough concentrations—BSA profiles according to the BSA-based dose regimen. The target range of trough concentrations, 5–15 ng/mL, is presented as a red dashed line.

Monte Carlo simulation based on the final PK model was performed to explore the optimal dose regimen in the population of FCD patients according to BSA, which influences everolimus concentration. Dosages were simulated based on BSA as follows: 3 mg/m^2^, 4.5 mg/m^2^, 5 mg/m^2^, 6 mg/m^2^, 7 mg/m^2^, and 9 mg/m^2^. The simulated concentration–time courses at steady state when BSA is 0.5 m^2^, 0.7 m^2^, 1 m^2^, 1.5 m^2^, 1.7 m^2^, and 2 m^2^ are presented in Supplementary Figures S1–S4. The target range of everolimus trough concentration in FCD was assumed to be from 5 to 15 ng/mL, according to a previous clinical trial on TSC (Franz et al., 2013). Figure 3 describes the simulated trough concentrations based on the BSA level and various BSA-based dosage regimens after administering everolimus once a day for 2 weeks, which is enough time to reach the steady state. The target range 5–15 ng/mL is presented as a red dashed line, and the efficacious dose regimens that allow the trough concentrations to achieve the target range were explored according to each baseline of BSA. Furthermore, we decided the optimal initial doses by both mean trough concentrations and % of target concentrations as 60%. Based on the simulation results, Table 3 suggests an optimal initial dose of everolimus by the BSA range, and this can guide the efficacious dose for FCD individual patients according to their BSA value.

**TABLE 3:** Optimal initial dose of everolimus by the BSA range.

BSA (m^2^)	Initial dose regimen
**0.5≤BSA< 1**	7–9 mg/m^2^ for the initial dose
**1.5 ≤BSA**	6–7 mg/m^2^ for the initial dose

## 4 Discussion

The dosing regimen of everolimus and the target range of trough concentrations for treating seizures associated with FCD have not been decided yet, and a population pharmacokinetic model of everolimus in FCD patients was explored to optimize the dosage regimen. As a result, the model was described as a one-compartmental model with first-order absorption, and the final model revealed that the clearance of everolimus increased linearly as BSA increased. Target concentrations and clinical endpoints of everolimus in FCD were not determined, and we used those of TSC. This is the first study to develop a population pharmacokinetic model and suggest the optimal dosage of everolimus in patients with FCD.

The population pharmacokinetic model of everolimus for FCD patients is developed as a one-compartment model, different from other population PK models of everolimus, which are two-compartment models (Fouladi et al., 2007; Dirk Jan et al., 2012; Atsuko Tanaka et al., 2016; de Wit et al., 2016; Combes et al., 2018; Ter Heine et al., 2018). Only one study reported PK in FCD patients, reporting a two-compartment model with a similar CL (20.0 L/h) and a larger Vd (V2 219 L and V3 335.7 L) (Combes et al., 2018), where the CL was similar to that in our study, and the Vd was larger than that in our study. The difference in Vd may be due to differences in the study population, with the age of the subjects in Combes et al.’s study ranging from 19 to 74 years. Some studies reported on cancer and transplant patients. One study conducted on liver transplant patients reported that everolimus follows a one-compartment model, with a smaller CL and a similar Vd to this study, with covariates of body weight, total daily dose, fluconazole concomitant administration, eGFR, and sex (Itohara et al., 2022). Heine et al. developed a mechanistic two-compartment model for both transplant and cancer patients. As a result, a much higher CL (364 L/h) and a larger Vc (176 L) and Vp (577 L) were reported, and the dosage regimen was recommended according to the therapeutic ranges of each disease (Ter Heine et al., 2018). As described previously, different studies have shown different PK results, and more studies are needed to confirm trends, especially in patients with FCD.

Because the time points of sampling were sparse in this study, many assumptions regarding parameters were required to fit the data to the two-compartmental model. Therefore, a one-compartmental model was preferred for simplicity. The inter-individual variability of the rate constant of absorption (ka) was fixed at zero because there was not much information about the absorption phase from our data. The estimate of ka in this population was 0.585 h^−1^ in the final model, with the assumption that absorption rates are the same among the patients. This estimate is lower than that of other populations, which ranged from 0.647 h^−1^ to 11.087 h^−1^. Combes et al. reported the rate constant of absorption as 10.8 h^−1^ from the population pharmacokinetic model of everolimus in patients with seizures associated with TSC (Combes et al., 2018). The difference of rate constants between this study and the study of Combes might have resulted from differences in the range of age and the control of food effect (Kovarik et al., 2002).

From the simulation in this study, the initial dose of everolimus for FCD patients with refractory seizures was suggested by individual BSA. The optimal doses were found from the result of simulations by the BSA of 0.5 m^2^, 0.7 m^2^, 1 m^2^, 1.5 m^2^, 1.7 m^2^, and 2 m^2^. For FCD patients whose BSAs are less than 1 m^2^, 7–9 mg/m^2^ of everolimus is recommended. For patients with BSA between 1 and 2 m^2^, 6–7 mg/m^2^ of everolimus is recommended. The initial recommended doses are higher than those of our study, and this means that different dosage regimens should be considered in FCD patients. To suggest a practical dose regimen for FCD patients by BSA, the available strengths of everolimus should be considered; therefore, the actual dose which is obtained by the multiplication of BSA should be rounded to be useful in practice.

The recommended dosing regimens for FCD patients are similar to those for TSC patients, considering the correlation between age and BSA (Andre et al., 2016). The effect of concomitant use of CYP3A4/P-gp inducers was not analyzed in this study because of insufficient numbers of patients who have been administered strong CYP3A4/P-gp inducers. However, BSA is expected to enable more precise dosing for individuals such as obese children when compared to an only age-based dosing regimen.

There are some limitations in this study. First, it was not possible to estimate the inter-individual variability of the absorption rate and the effect of concomitant use of CYP3A4/P-gp inducers/inhibitors on the pharmacokinetics of everolimus because the number of samples was not enough. To suggest a more optimal dose regimen of everolimus for FCD patients, the reference range of target therapeutic concentration needs to be set first. The assumption that the therapeutic range of everolimus in FCD patients is similar to that of TSC patients was required to recommend the optimal dose in this study. Moreover, it is expected that the limit of maximum concentrations should be decided, and more precise dose regimens can be suggested for the FCD patient population using safety information from clinical studies.

## 5 Conclusion

The population pharmacokinetic model of everolimus for patients with refractory seizures associated with FCD was built as a one compartment model with first-order absorption and elimination. The linear effect of BSA on clearance was included in the final model. Based on the developed population pharmacokinetic model, the optimal dose regimen of everolimus for individuals with FCD was recommended by considering the BSA and strength of formulations in practice.

## Data Availability

Raw data with personal information removed can be provided upon request.
