# Supplementary material for: Population pharmacokinetics of everolimus in patients with seizures associated with focal cortical dysplasia
Source: Front Pharmacol. 2023 Nov 24;14:1197549. doi: 10.3389/fphar.2023.1197549 (PMC10704167; doi:10.3389/fphar.2023.1197549)
Supplement: Supplementary file 1 [file DataSheet1.PDF]

1. The inclusion/exclusion criteria

The inclusion criteria of the participants are as follows:

- Patients from 4 to 40 years of age
- Patients with focal cortical dysplasia (FCD) type II
- Patients with refractory seizures despite of using more than two anti-epileptic drugs
- Patients who experienced more than three times of seizures per month over two months despite of using more than one anti-epileptic drug for three months before the screening period
- Patients with no responses to standard treatments of seizure such as vagus nerve stimulation or dietary treatment during baseline phase
- Patients who experienced at least more than three times of seizures during baseline phase
- Patients who agreed to participate in this trial by providing fully informed consent with their guardians' agreement

The exclusion criteria are as follows:

- Patients who need in-hospital treatment for disease not relevant to focal cortical dysplasia
- Patients who are pregnant or have a plan to pregnancy
- Patients experiencing seizures caused by other factors not focal cortical dysplasia
- Patients who have immune deficient diseases
- Patients who have taken mTOR inhibitor including everolimus before
- Patients who have not been followed up for one year lately
- Patients whose EEG result is normal
- Patients who stop taking anti-epileptic drugs or adjust dosage of drugs for themselves

- Patients who are positive to HBV Ag
- Patients who are vaccinated during baseline phase
- Patients who are sensitive to rapamycin derivatives such as everolimus and sirolimus and the other ingredients of this drug
- Patients who have galactose intolerance, lactose deficiency, or glucose-galactose malabsorption

2. The list of CYP3A4 inducer and inhibitor included in this analysis

Table S1. List of co-administered CYP 3A4 inducers and inhibitors

	**CYP 3A4 inducers**	**CYP 3A4 inhibitors**
Strong	Carbamazepine<br>Phenytoin	Clarithromycin
Moderate	Phenobarbital	Cyclosporine
Weak	Rufinamide<br>Topiramate<br>Clobazam<br>Perampanel<br>Oxcarbazepine	Valproic acid<br>Perampanel<br>Ranitidine

CYP, cytochrome P450 enzyme

3. Scenarios of simulation

Table S2. Scenarios of simulation

Scenario number	Dose (mg/m^2)	Body surface area (m^2)	Actual amount (mg)
**1**	3	0.5	1.5
**2**	3	1	3
**3**	3	1.5	4.5
**4**	3	1.7	5.1
**5**	3	2	6
**6**	4.5	0.5	2.25
**7**	4.5	1	4.5
**8**	4.5	1.5	6.75
**9**	4.5	1.7	7.65
**10**	4.5	2	9
**11**	5	0.5	2.5
**12**	5	1	5
**13**	5	1.5	7.5
**14**	5	1.7	8.5
**15**	5	2	10
**16**	7	0.5	3.5
**17**	7	1	7
**18**	7	1.5	10.5
**19**	7	1.7	11.9
**20**	7	2	14
**21**	9	0.5	4.5
**22**	9	1	9
**23**	9	1.5	13.5
**24**	9	1.7	15.3
**25**	9	2	18
**26**	3	0.7	2.1
**27**	4.5	0.7	3.15
**28**	5	0.7	3.5
**29**	6	0.7	4.2
**30**	7	0.7	4.9
**31**	6	0.5	3
**32**	6	1	6
**33**	6	1.5	9
**34**	6	1.7	10.2
**35**	6	2	12

4. Simulated PK profiles by BSA baseline and BSA-based dose regimen

PK profiles by BSA were simulated after administered everolimus once a day for 2 weeks based on various BSA-based dose regimen. The target trough concentration range, 5 – 15 ng/mL, was presented as red dashed lines in the figures.

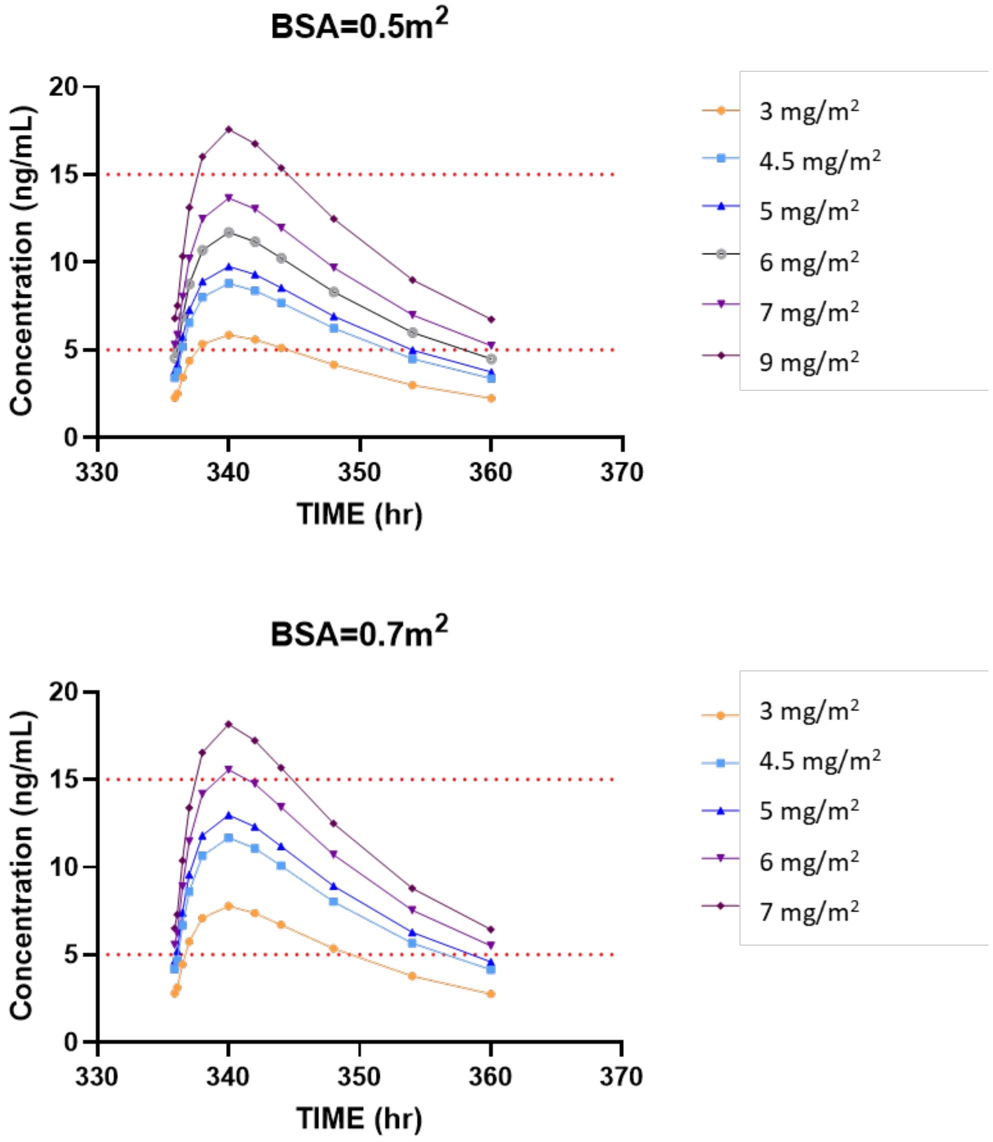

Figure S1. Concentration-time course at steady state by dosage regimens in BSA of 0.5 m^2 and 0.7 m^2

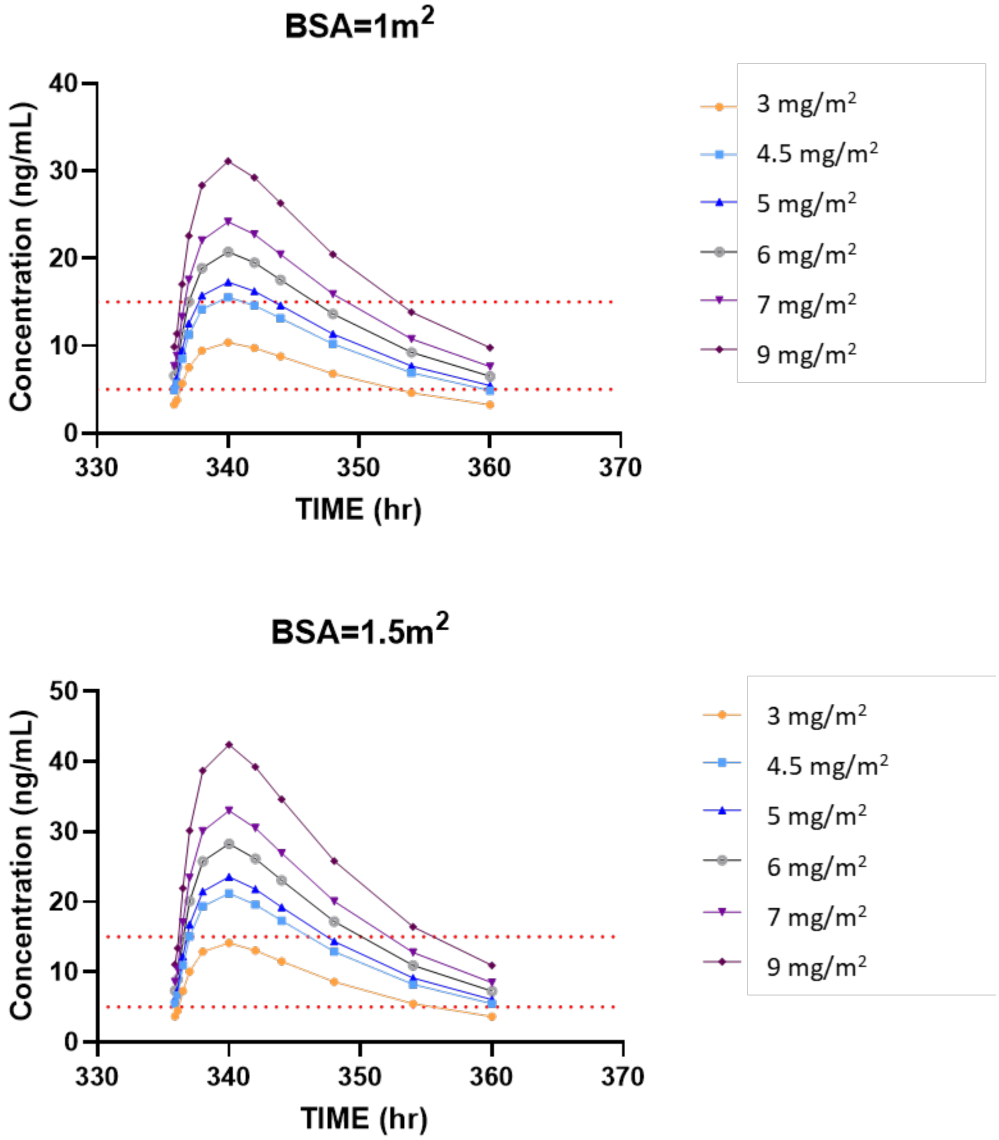

Figure S2. Concentration-time course at steady state by dosage regimens in BSA of 1 m^2 and 1.5 m^2

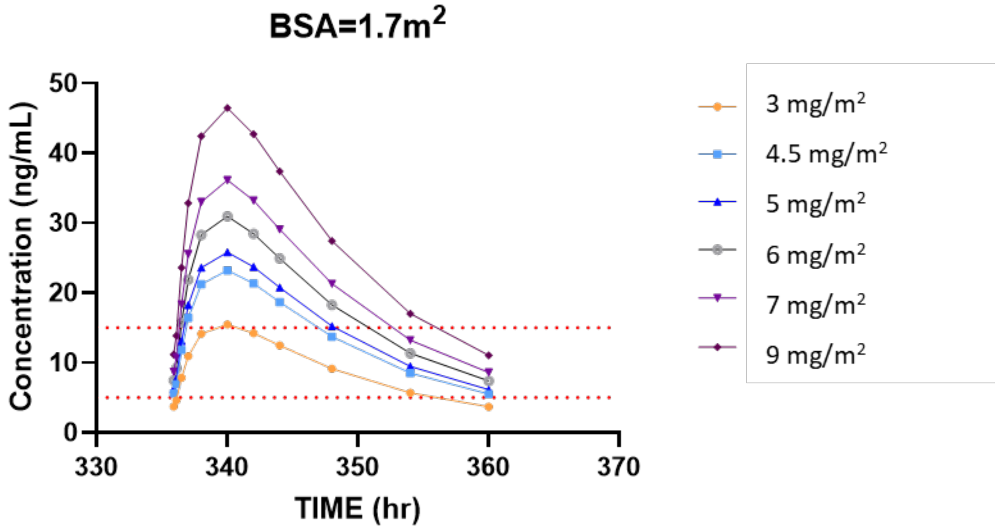

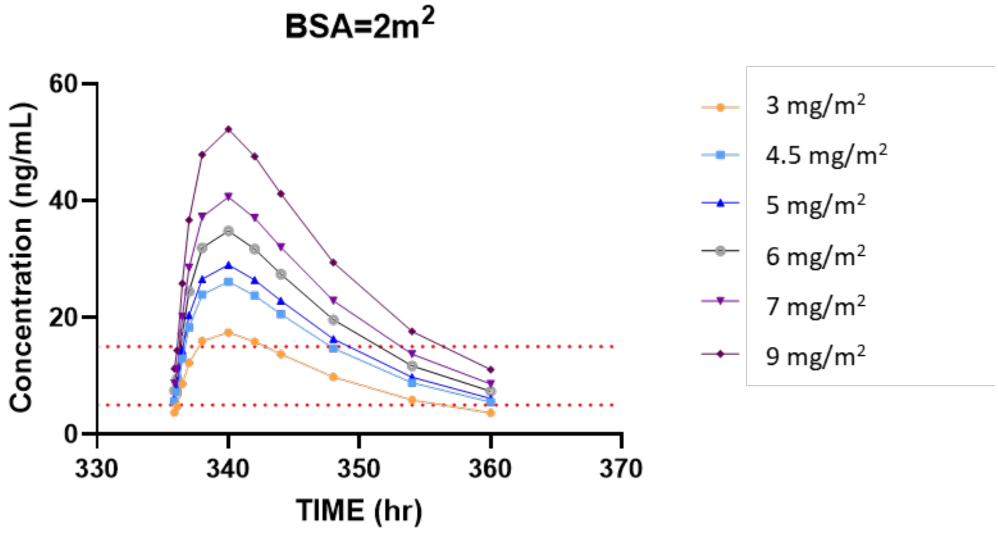

Figure S3. Concentration-time course at steady state by dosage regimens in BSA of 1.7 m^2 and 2 m^2

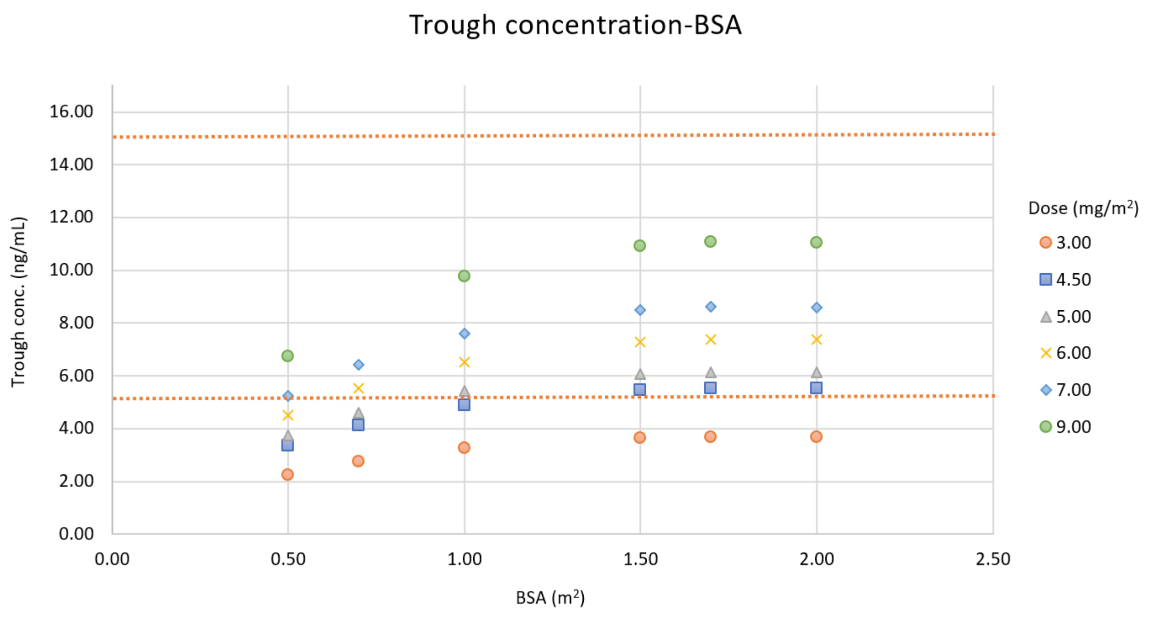

Trough concentration-BSA
Trough conc. (ng/mL)
BSA (m2)
Dose (mg/m2)
3.00
4.50
5.00
6.00
7.00
9.00

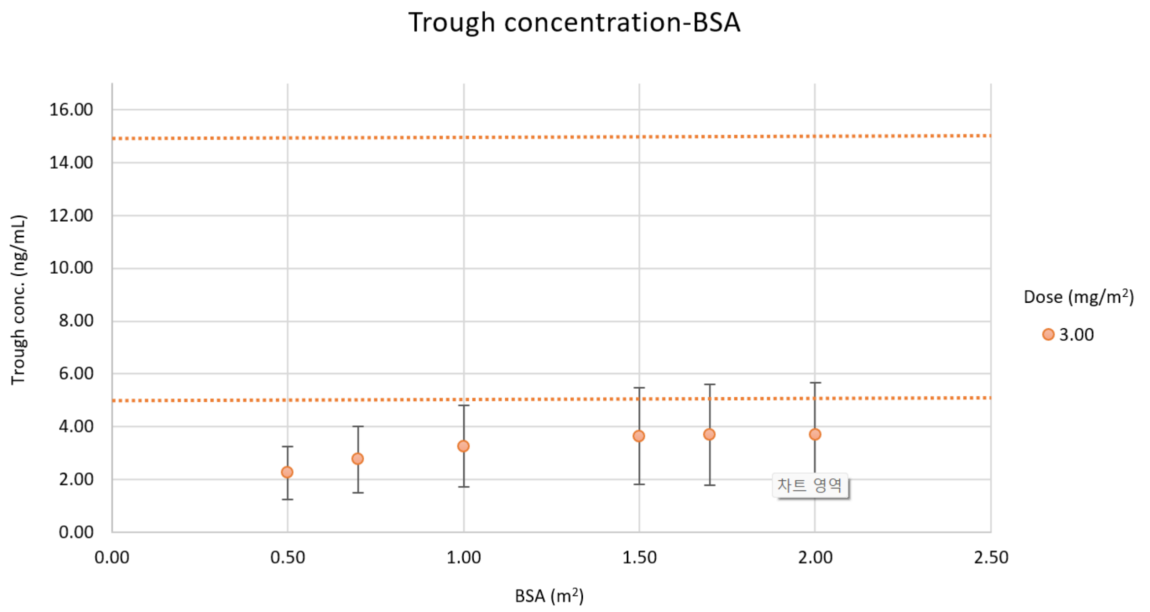

Trough concentration-BSA
Trough conc. (ng/mL)
BSA (m2)
Dose (mg/m2)
3.00
차트 영역

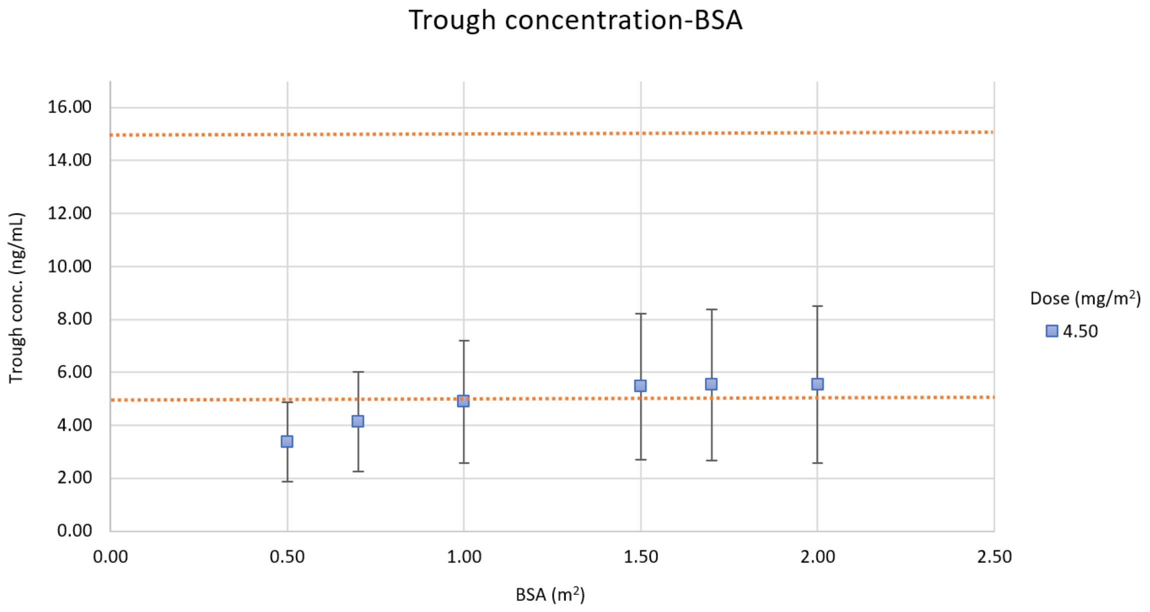
Trough concentration-BSA
Trough conc. (ng/mL)
16.00
14.00
12.00
10.00
8.00
6.00
4.00
2.00
0.00
0.00
0.50
1.00
1.50
2.00
2.50
BSA (m2)
Dose (mg/m2)
4.50

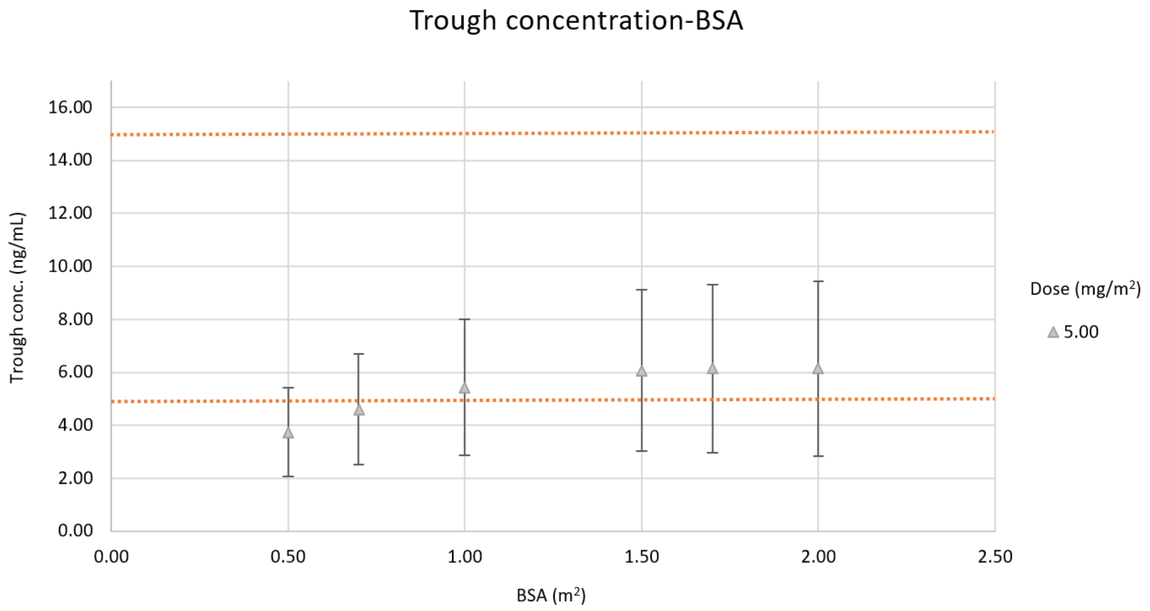
Trough concentration-BSA
Trough conc. (ng/mL)
16.00
14.00
12.00
10.00
8.00
6.00
4.00
2.00
0.00
0.00
0.50
1.00
1.50
2.00
2.50
BSA (m2)
Dose (mg/m2)
5.00

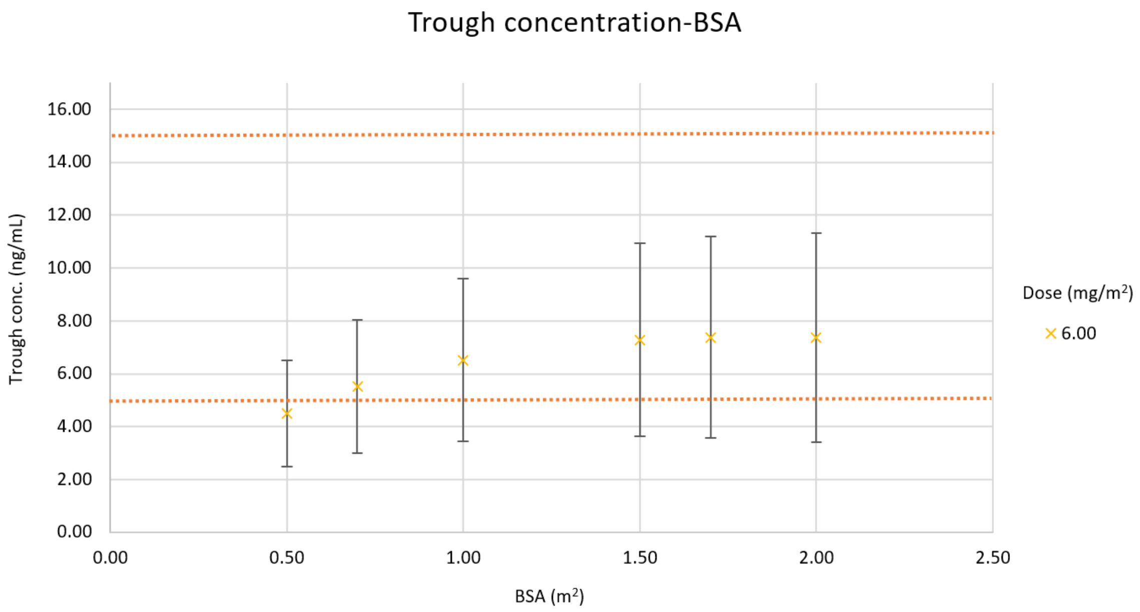
Trough concentration-BSA
Trough conc. (ng/mL)
16.00
14.00
12.00
10.00
8.00
6.00
4.00
2.00
0.00
0.00
0.50
1.00
1.50
2.00
2.50
BSA (m2)
Dose (mg/m2)
6.00

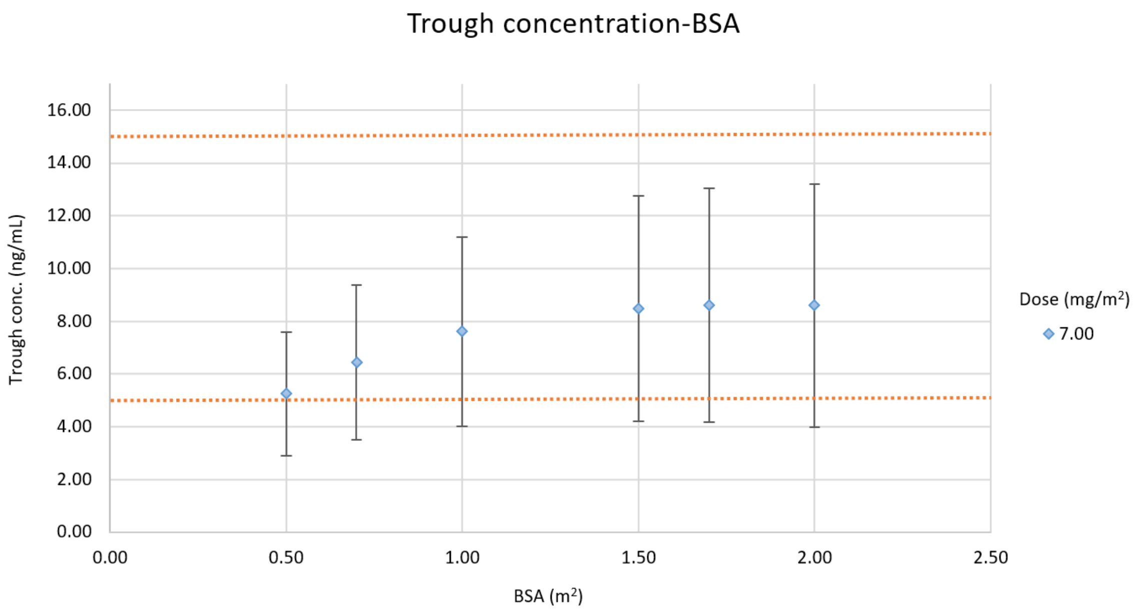
Trough concentration-BSA
Trough conc. (ng/mL)
16.00
14.00
12.00
10.00
8.00
6.00
4.00
2.00
0.00
0.00
0.50
1.00
1.50
2.00
2.50
BSA (m2)
Dose (mg/m2)
7.00

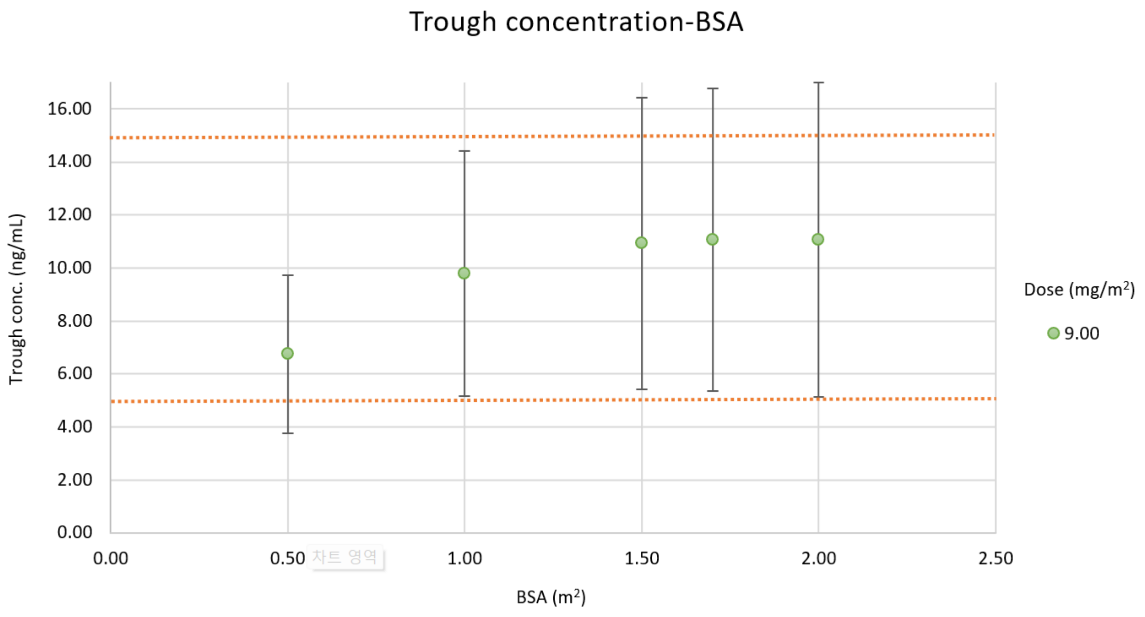

Figure S4. Simulated trough concentrations based on body surface area (BSA).

Data are expressed as mean (dot) and standard deviation (error bars).

Dot lines are target range of everolimus.

Table S3. Simulated trough concentrations and % of target range (5 – 15 ng/mL)

Body surface area (m^2)	Dose (mg/m^2)	Mean trough concentrations (ng/mL)	Standard devication	% of target range (5-15 ng/mL)
0.5	3	2.25	1.00	1.2
0.5	4.5	3.37	1.50	13
0.5	5	3.75	1.66	20.8
0.5	6	4.50	2.00	37.3
0.5	7	5.25	2.33	50.5
0.5	9	6.75	3.00	70.4
0.7	3	2.76	1.25	4.6
0.7	4.5	4.14	1.88	28.4
0.7	5	4.60	2.09	39.1
0.7	6	5.52	2.51	53
0.7	7	6.43	2.93	65.7
1	3	3.26	1.54	12.4
1	4.5	4.89	2.31	44.9
1	5	5.43	2.57	51.8
1	6	6.52	3.08	66.2
1	7	7.61	3.59	77.7
1	9	9.78	4.62	98.6
1.5	3	3.64	1.83	20.8
1.5	4.5	5.46	2.75	51.3
1.5	5	6.07	3.05	59.1
1.5	6	7.28	3.66	73.5
1.5	7	8.50	4.27	85.8
1.5	9	10.93	5.49	8.7
1.7	3	3.69	1.90	21.7
1.7	4.5	5.53	2.86	52.1
1.7	5	6.15	3.17	59.8
1.7	6	7.38	3.81	73.4
1.7	7	8.61	4.44	85.4
1.7	9	11.07	5.71	9.3
2	3	3.69	1.98	22.2
2	4.5	5.53	2.96	51.3
2	5	6.14	3.29	59.4
2	6	7.37	3.95	73.1
2	7	8.60	4.61	86
2	9	11.06	5.93	8.1
